# A new one-parameter lifetime distribution and its regression model with applications

**DOI:** 10.1371/journal.pone.0246969

**Published:** 2021-02-19

**Authors:** M. S. Eliwa, Emrah Altun, Ziyad Ali Alhussain, Essam A. Ahmed, Mukhtar M. Salah, Hanan Haj Ahmed, M. El-Morshedy

**Affiliations:** 1 Department of Mathematics, College of Science, Majmaah University, Majmaah, Saudi Arabia; 2 Department of Mathematics, Faculty of Science, Mansoura University, Mansoura, Egypt; 3 Department of Mathematics, Bartin University, Bartin, Turkey; 4 Department of Administrative and Financial Sciences, Taibah University, Community College of Khyber, Medina, Saudi Arabia; 5 Department of Mathematics, Sohag University, Sohag, Egypt; 6 Department of Basic Science, Preparatory Year Deanship, King Faisal University, Hofuf, Al-Ahsa, Saudi Arabia; 7 Department of Mathematics and Statistics, College of Science and Humanities in Al-Kharj, Prince Sattam bin Abdulaziz University, Al-Kharj, Saudi Arabia; 8 Department of Mathematics and Statistics, Faculty of Science, Mansoura University, Mansoura, Egypt; Tongii University, CHINA

## Abstract

Lifetime distributions are an important statistical tools to model the different characteristics of lifetime data sets. The statistical literature contains very sophisticated distributions to analyze these kind of data sets. However, these distributions have many parameters which cause a problem in estimation step. To open a new opportunity in modeling these kind of data sets, we propose a new extension of half-logistic distribution by using the odd Lindley-G family of distributions. The proposed distribution has only one parameter and simple mathematical forms. The statistical properties of the proposed distributions, including complete and incomplete moments, quantile function and Rényi entropy, are studied in detail. The unknown model parameter is estimated by using the different estimation methods, namely, maximum likelihood, least square, weighted least square and Cramer-von Mises. The extensive simulation study is given to compare the finite sample performance of parameter estimation methods based on the complete and progressive Type-II censored samples. Additionally, a new log-location-scale regression model is introduced based on a new distribution. The residual analysis of a new regression model is given comprehensively. To convince the readers in favour of the proposed distribution, three real data sets are analyzed and compared with competitive models. Empirical findings show that the proposed one-parameter lifetime distribution produces better results than the other extensions of half-logistic distribution.

## 1 Introduction

There are many distributions in the statistics literature. However, each data sings its song. Therefore, finding a suitable probability distribution for each data is an important issue to catch all parts of the song. In the data analysis process, we encounter two types of data. These are discrete and continuous data sets. It is necessary to decide on the appropriate distribution for each data type. To increase the accuracy in data modeling, the researchers have introduced flexible distributions for both discrete and continuous cases. However, the aim of the presented manuscript complies on the continuous probability distributions. [1–17] have introduced flexible continuous distri-butions based on the T-X family of [18]. In these studies, researchers have generalized the baseline distributions by adding one or more additional shape parameters to increase the flexibility of the baseline distributions. The generalization of the exponential, Weibull, generalized half-normal and Lindley distributions have gained attention by researchers because of their importance in lifetime and reliability modeling. Apart from these distributions, the Half-logistic (HL) is also an important distribution for reliability analysis and increased its popularity in recent years. The detail information on HL distribution can be found in [19]. The probability density function (pdf) of the HL distribution is

$$\begin{array}{c} g\left(x\right)=\frac{2e^{-x}}{{\left(1+e^{-x}\right)}^{2}};\;\;x>0. \end{array}$$ (1)

The corresponding cumulative distribution function (cdf) to (1) is

$$\begin{array}{c} G\left(x\right)=\frac{1-e^{-x}}{1+e^{-x}};\;\;x>0. \end{array}$$ (2)

The several generalizations of (1) have been introduced such as exponential half-logistic additive model by [20], extended half-logistic distribution by [21], type-I half-logistic distribution by [22], exponentiated half-logistic-G by [23], transmuted half-logistic distribution by [24] and half-logistic inverse Rayleigh by [25].

In this study, we use the odd Lindley-G family of [8] to introduce a new generalization of HL distribution. The reason for the use of odd Lindley-G (OLi-G) family is that it has only one additional shape parameter. It means that the proposed distribution will also have only one parameter. Increasing the parameter space of the probability distributions causes a problem in the estimation step. The parsimony rule says the best model is a model which requires less assumptions and parameters. Therefore, it is more preferred to study with less parameters and less complexity. However, more complex models are still needed to model the different characteristics of the data sets. The cdf and pdf of the OLi-G family are given, respectively, by

$$\begin{array}{c} F\left(x;\lambda ,\eta \right)=1-\frac{\lambda +\bar{G}\left(x;\eta \right)}{\left(1+\lambda \right)\bar{G}\left(x;\eta \right)}e^{-\lambda \frac{G(x;\eta )}{\bar{G}\left(x;\eta \right)}};\;x\geq 0, \end{array}$$ (3)

$$\begin{array}{c} f\left(x;\lambda ,\eta \right)=\frac{\lambda^{2}g\left(x;\eta \right)}{\left(1+\lambda \right)\bar{G}{\left(x;\eta \right)}^{3}}e^{-\lambda \frac{G(x;\eta )}{\bar{G}\left(x;\eta \right)}};\;x\geq 0, \end{array}$$ (4)

where $\bar{G}\left(x;\eta \right)=1-G\left(x;\eta \right)$ represents the reliability function. As seen from (3) and (4), the OLi-G family has simple forms for its pdf and cdf. This property of the OLi-G family has attracted us to use it for a new generalization of the HL distribution.

The remaining parts of the presented study are arranged as follows. In Section 2, we define the OLiHL distribution and studied its important statistical properties. The parameter estimation problem of the OLiHL distribution is discussed in Section 3 with four different estimation methods. Section 4 deals with a new location-scale regression model based on the OLiHL distribution for modeling extremely left-skewed lifetime variables with covariates. In Section 5, the finite sample performance of the different estimation methods is compared with the simulation study. In Section 6, two real data sets are analyzed with the proposed models. The discussions on the empirical results are given in Section 7. The conclusions are given in Section 8.

## 2 The OLiHL distribution

Now, we introduce a new generalization of the HL distribution, OLiHL distribution, by inserting (2) in (3). Let the random variable *X* follows an OLiHL distribution if its cdf is given by

$$\begin{array}{c} F\left(x;\lambda \right)=1-\frac{\lambda e^{x}+\lambda +2}{2(\lambda +1)}e^{-\frac{\lambda }{2}\left(e^{x}-1\right)};\;x>0, \end{array}$$ (5)

where λ > 0. The pdf of the OLiHL distribution is

$$\begin{array}{c} f\left(x;\lambda \right)=\frac{\lambda^{2}\left(1+e^{x}\right)}{4(1+\lambda )}e^{-\frac{\lambda }{2}\left(e^{x}-1\right)+x};\;x>0. \end{array}$$ (6)

The corresponding hazard rate function (hrf) to (6) is

$$\begin{array}{c} h\left(x;\lambda \right)=\frac{\lambda^{2}\left(1+e^{x}\right)}{2[\lambda \left(1+e^{-x}\right)+2e^{-x}]};\;x>0. \end{array}$$ (7)

**Fig 1:**
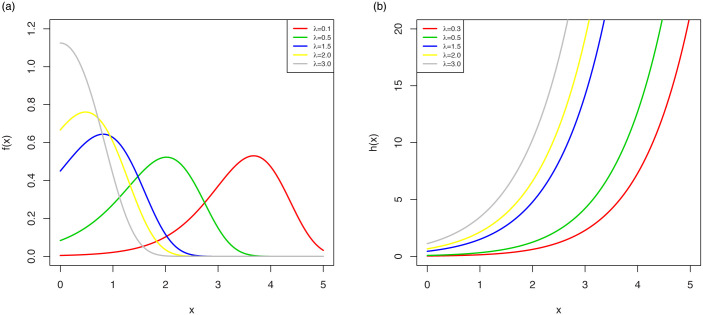
The pdf (left) and hrf (right) plots of the OLiHL distribution.

The possible shapes of the pdf and hrf are displayed in Fig 1 which shows that the OLiHL distribution could be a proper distribution for right-skewed and unimodal data sets with increasing hrf shapes.

Following the work of [8], the physical interpretation of the OLiHL distribution can be given as follows. Let the random variable *X* represents lifetimes of individuals following the HL distribution, given in 1. Consider that we are interested to model the odds that an individual dies before a given time *X* which is given by *F*(*x*)/(1 − *F*(*x*)) where *F*(*x*) is the cdf of the HL distribution, given in 2. Let consider that we requires to model the randomness of the odds by the random variable *Y*, follows the Lindley distribution. Then, we can write

$$\begin{array}{ccc} \text{Pr}(Y<y) & = & F_{Y}(\frac{F(x)}{1-F(x)})=F_{Y}(\frac{1-e^{-x}}{2e^{-x}})\\ & = & 1-\frac{\lambda e^{x}+\lambda +2}{2(\lambda +1)}e^{-\frac{\lambda }{2}\left(e^{x}-1\right)} \end{array}$$ (8)

which is identical to 5.

**Fig 2:**
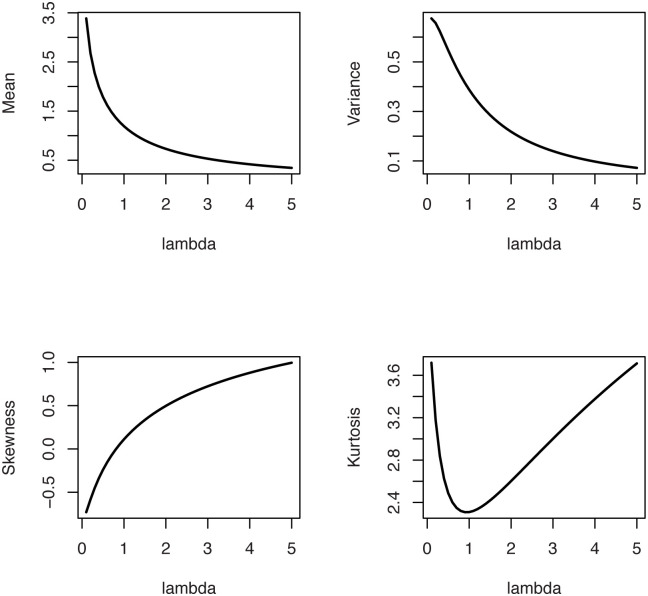
The mean, variance, skewness and kurtosis measures of the OLiHL distribution.

The statistical properties of the OLiHL distribution such as raw and central moments, incomplete moments, generating functions can be obtained following the results of [8]. [8] introduced the general representation of the OLi-G family for all baseline distributions. Therefore, using the HL distribution as a baseline distribution of the OLi-G family, one can easily obtain the required expansions for the moments and generating functions of the OLiHL distribution. Since these expansions are accessible in the work of [8], we omit them in this study. However, the numerical values of the mean, variance, skewness, and kurtosis measures of the OLiHL distribution are computed and displayed in Fig 2 which shows that the mean and variance of the OLiHL distribution are decreasing function of the parameter λ. The OLiHL distribution can be left or right-skewed. The distribution is nearly symmetric when the parameter λ ≅ 0.81. The OLiHL distribution has also playtokurtic and leptokurtic shapes based on its kurtosis values.

The quantile function (qf) is an important function to generate random variables from a specific continuous probability distribution. The qf is a solution of *F*(*x*) = *u* equation for *x*, then *F*(*u*)^−1^ = *x* represents the qf which is denoted as *Q*(*u*). The qf of the OLiHL distribution is

$$\begin{array}{c} Q\left(u\right)=\text{ln}\;\{\frac{-2}{\lambda }[1+W_{-1}((1+\lambda )\left(u-1\right)e^{-(1+\lambda )})]-1\}, \end{array}$$ (9)

where *u* ∈ (0, 1) and *W*_−1_ is the negative branch of the Lambert-W function. The random variables from OLiHL distribution can be generated by using the qf in (9). To do this, the following algorithm steps can be implemented.

1. Set the parameter λ.
2. Generate *u* ∼ *U*(0, 1).
3. Using the generated *u*, calculate
  $$\begin{array}{c} Q\left(u\right)=\text{ln}\;\{\frac{-2}{\lambda }[1+W_{-1}((1+\lambda )\left(u-1\right)e^{-(1+\lambda )})]-1\} \end{array}$$ (10)
4. Repeat the steps 2 and 3 *N* times.

## 3 Different estimation methods

The parameter estimation problem of the OLiHL distribution is discussed in detail. The four-parameter estimation methods are used to estimate the unknown parameter of the OLiHL distribution. The rest of this section is devoted to the mathematical framework of these estimation methods.

### 3.1 Maximum likelihood estimation

Let *X*_1_, *X*_2_, …, *X*_*n*_ come from the OLiHL distribution unknown parameter λ. The maximum likelihood estimation (MLE) of λ, say ${\hat{\lambda }}_{MLE}$, is obtained by maximizing the following log-likelihood equation

$$\begin{array}{c} \begin{array}{c} \ell \left(\lambda \right)=\sum_{i=1}^{n}\text{log}\;f\left(x_{i};\lambda \right)\\ =2n\;\text{ln}\;\lambda -n\;\text{ln}\left(4+4\lambda \right)+\sum_{i=1}^{n}\;\text{ln}\left(1+e^{x_{i}}\right)\\ -\frac{\lambda }{2}\sum_{i=1}^{n}\left(e^{x_{i}}-1\right)+\sum_{i=1}^{n}x_{i}. \end{array} \end{array}$$ (11)

Alternatively, the direct maximization of (11) is equal to solve the first derivative of (11) for the parameter λ for zero, which is given by

$$\begin{array}{c} \frac{n(2+\hat{\lambda })}{\hat{\lambda }\left(1+\hat{\lambda }\right)}-\frac{1}{2}\sum_{i=1}^{n}\left(e^{x_{i}}-1\right)=0. \end{array}$$

Since there is no explicit solution of this equation for λ, one should use iterative methods to solve it. More information on MLE method can be found in [26, 27].

### 3.2 Least square and weighted least square estimations

The least-square estimation (LSE) and weighted LSE (WLSE) methods are based on the minimization of the distance between the empirical and theoretical distribution functions. Assume that *x*_(1)_, *x*_(2)_, ⋯, *x*_(*n*)_ represents the ordered sample of a random sample with known cdf, *F*(⋅). Let *F*(*X*_(*j*)_) represents the *j*th order statistics from standard uniform distribution, *U*(0, 1). It is well-known that the *j*th order statistics of *U*(0, 1) is distributed as Beta(*j*, *n* − *j* − 1) (see [28]). So, we have

$$\begin{array}{c} E[F(X_{\left(j\right)})]=\frac{j}{n+1}, \end{array}$$ (12)

$$\begin{array}{c} \text{Var}[F(X_{\left(j\right)})]=\frac{j(n-j+1)}{{\left(n+1\right)}^{2}(n+2)}. \end{array}$$ (13)

The LSE of the parameter of the OLiHL distribution is obtained by minimizing the following function.

$$\begin{array}{c} \text{LSE}\;(\lambda )=\sum_{j=1}^{n}{\left[F(X_{\left(j\right)}|\lambda )-\frac{j}{n+1}\right]}^{2}, \end{array}$$ (14)

where *F*(*X*_(*j*)_|λ) is the cdf of the OLiHL distribution, given in (5). Substituting *F*(*X*_(*j*)_|λ) with (5) in (14), we have

$$\begin{array}{c} \sum_{j=1}^{n}(1-\frac{\lambda e^{x_{\left(j\right)}}+\lambda +2}{2(\lambda +1)}e^{-\frac{\lambda }{2}(e^{x_{\left(j\right)}}-1)}-\frac{j}{n+1}). \end{array}$$ (15)

The WLSE of the parameter of the OLiHL distribution is obtained by minimizing the following function.

$$\begin{array}{c} \begin{array}{c} \text{WLSE}(\lambda )=\sum_{j=1}^{n}w_{j}{\left[F(X_{\left(j\right)}|\lambda )-\frac{j}{n+1}\right]}^{2}. \end{array} \end{array}$$ (16)

where $w_{j}=\frac{1}{\text{Var}[F(X_{j})]}$. Substituting *F*(*X*_(*j*)_|λ) with (5) in (16), we have

$$\begin{array}{c} \begin{array}{c} \sum_{j=1}^{n}\frac{{\left(n+1\right)}^{2}(n+2)}{j(n-j+1)}\\ \times (1-\frac{\lambda e^{x_{\left(j\right)}}+\lambda +2}{2(\lambda +1)}e^{-\frac{\lambda }{2}(e^{x_{\left(j\right)}}-1)}-\frac{j}{n+1}) \end{array} \end{array}$$ (17)

### 3.3 Cramér-von Mises minimum distance estimation

Cramér-von Mises estimation (CVME) method is also based on the minimization of the distance between the empirical and theoretical distribution functions. The CVME and WLSE use different weighting functions and the biases CVME is less than those of WLSE. The WLSE of the parameter λ is obtained by minimizing the below function for the parameter λ.

$$\begin{array}{c} \text{CVME}\left(\lambda \right)=\frac{1}{12n}+\sum_{j=1}^{n}{\left[F(x_{\left(j\right)}|\lambda )-\frac{2j-1}{2n}\right]}^{2}. \end{array}$$ (18)

## 4 The log-OLiHL regression model

The log-location-scale regression models are popular models to analyze the censored response variable with some covariates. In the last decade, researchers have introduced flexible location-scale regression models to analyze the different characteristics of the data sets. The important papers on location-scale can be cited as follows: log-generalized odd log-logistic-Weibull regression model by [29], log-odd log-logistic Burr XII regression model by [30], log-odd log-logistic generalized half-normal regression model by [31]. These types of regression models were introduced based on the *Y* = log (*X*) transformation and suitable re-parametrization on the baseline distribution.

Now, we adopt this approach to the OLiHL distribution. Let the random variable *X* follows an OLiHL distribution with the parameter λ. Using the transformation *Y* = log(*X*) and adding location and scale parameters, we have

$$\begin{array}{ccc} f(y;\lambda ,\mu ,\sigma ) & = & \frac{\lambda^{2}}{\sigma (4\lambda +4)}[\text{exp}\;(\text{exp}\;[\frac{y-\mu }{\sigma }])+1]\\ & \times & \text{exp}\;[\frac{y-\mu }{\sigma }+\text{exp}\;[\frac{y-\mu }{\sigma }]-\frac{\lambda (\text{exp}\;(\text{exp}\;[\frac{y-\mu }{\sigma }])-1)}{2}], \end{array}$$ (19)

where *y* ∈ ℜ, *μ* ∈ ℜ and *σ* > 0. The parameters *μ* and *σ* are the location and scale parameters of the LOLiHL distribution. Hereafter, the pdf in Eq (19) is called as log-OLiHL (LOLiHL) distribution. The corresponding cdf and survival function (sf) to Eq (19) are given, respectively, by

$$\begin{array}{c} \begin{array}{c} S(y)=\frac{\lambda +\lambda \;\text{exp}\;(\text{exp}\;[\frac{y-\mu }{\sigma }])+2}{2\lambda +2}\\ \times \text{exp}\;(-\frac{\lambda }{2}(\text{exp}\;(\text{exp}\;[\frac{y-\mu }{\sigma }])+1)). \end{array} \end{array}$$ (20)

**Fig 3:**
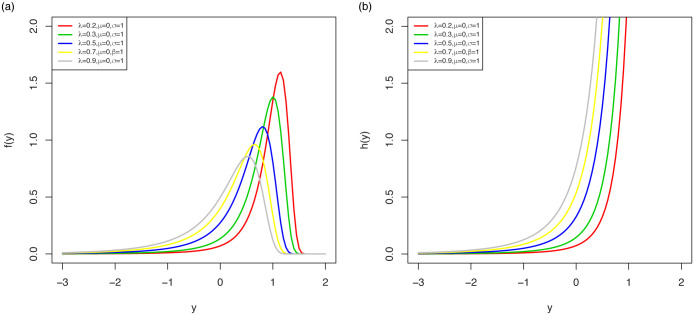
The pdf (left) and hrf (right) plots of LOLiHL distribution.

The pdf shapes of the LOLiHL distribution are displayed in Fig 3. As seen from these figures, the LOLiHL distribution has increasing failure rate and can be used to analyze the left-skewed lifetimes.

Consider the following regression model,

$$\begin{array}{c} y_{i}=x_{i}^{T}\beta +\sigma z_{i}, \end{array}$$ (21)

where the response variable *y*_*i*_ has the density given in Eq (19). The covariates are linked to location of *y*_*i*_ with identity link function $\mu_{i}=x_{i}^{T}\beta$ where **X** = (**x**_1_, **x**_2_, …, **x_N_**)^T^ is the model matrix consists of the observations of independent variables and ***β*** = (*β*_0_, *β*_1_, …, *β*_*k*_) is the unknown regression coefficients. Let the random sample *y*_1_, *y*_2_, …, *y*_*n*_ follow a LOLiHL distribution and the response variable is defined as *y*_*i*_ = min(*x*_*i*_, *c*_*i*_) where *c*_*i*_ is the censoring time and *x*_*i*_ is the observed lifetime. Assume that the censoring times and lifetimes are independent.

Let *F* and *C* are the sets representing the observed lifetimes and censoring times. The general formulation of the log-likelihood function for the model given in (21) is given by

$$\begin{array}{c} \ell (\theta )=\sum_{i\in F}\text{log}\;[f(y_{i})]+\sum_{i\in C}\text{log}\;[S(y_{i})] \end{array}$$ (22)

where ***θ*** is the unknown parameter vector, log[*f*(*y*_*i*_)] and log[*S*(*y*_*i*_)] are given in Eqs (19) and (20), respectively. Inserting Eqs (19) and (20) in Eq (22), we have following the log-likelihood function for the LOLiHL regression model

$$\begin{array}{c} \begin{array}{c} \ell (\theta )=r(1+\text{ln}(\frac{\lambda^{2}}{\sigma (4\lambda +4)}))\\ +2\sum_{i\in F}(\text{exp}\;[\frac{y_{i}-x_{i}^{T}\beta }{\sigma }])+\sum_{i\in F}(\frac{y_{i}-x_{i}^{T}\beta }{\sigma })\\ -\sum_{i\in F}2^{-1}\lambda (\text{exp}\;(\text{exp}\;[\frac{y_{i}-x_{i}^{T}\beta }{\sigma }])-1)\\ +\sum_{i\in C}\text{ln}(\frac{\lambda +\lambda \text{exp}\;(\text{exp}\;[\frac{y_{i}-x_{i}^{T}\beta }{\sigma }])+2}{2\lambda +2})\\ -\sum_{i\in C}\frac{\lambda }{2}(\text{exp}\;(\text{exp}\;[\frac{y_{i}-x_{i}^{T}\beta }{\sigma }])+1), \end{array} \end{array}$$

where *r* is the number of uncensored observations and ***θ*** = (λ, ***β***, *σ*) is the unknown parameter vector. The unknown parameter vector, ***θ***, is estimated by using the MLE method. The minus of the log-likelihood function is minimized by **optim** function of R software. The observed information matrix evaluated at $\hat{\theta }$ is used to obtain corresponding standard errors to construct asymptotic confidence intervals.

### 4.1 Residual analysis

Residual analysis is an important step of any regression analysis to check the sufficiency of the fitted model. If the fitted model is accurate for the data used, the residuals have to meet the distributional assumptions. Here, we used two kinds of residuals: modified deviance and martingale residuals. The martingale residuals under the LOLiHL regression model are given by

$$\begin{array}{c} r_{M_{i}}=\{\begin{array}{c} 1+\text{ln}(\begin{array}{c} \frac{\lambda +\lambda \text{exp}\;(u_{i})+2}{2\lambda +2}\\ \times \text{exp}\;(-\frac{\lambda }{2}(\text{exp}\;(u_{i})+1)) \end{array}),\;\text{if}\;i\in F\\ \text{ln}(\begin{array}{c} \frac{\lambda +\lambda \text{exp}\;(u_{i})+2}{2\lambda +2}\\ \times \text{exp}\;(-\frac{\lambda }{2}(\text{exp}\;(u_{i})+1)) \end{array}),\;\text{if}\;i\in C \end{array} \end{array}$$

where $u_{i}=\text{exp}\;\left(\left(y_{i}-x_{i}^{⊤}\beta \right)/\sigma \right)$. The modified deviance residual of LOLiHL model is

rDi={sign(rMi){−2[rMi+log(1−rMi)]}1/122,ifi∈Fsign(rMi){−2rMi}1/122ifi∈C

where $r_{M_{i}}$ is the martingale residual.

## 5 Simulation results

The relative performance of the MLE, LSE, WLSE, and CVME methods are discussed. The below simulation steps are carried out.

1. Set the simulation replication *N* = 1000 and the parameter value λ = 0.1
2. Generate random samples from OLiHL with sample sizes *n* = 20, 25, 30, …, 100
3. For each generated sample, obtain the MLE, LSE, WLSE and CVME of the parameter λ, say ${\hat{\lambda }}_{j}$ for *j* = 1, 2, …, 1000.
4. Using the estimated value of λ and true parameter value, calculate the biases and means square errors (MSEs) for each parameter estimation methods by using the below equations.
  $$\begin{array}{c} Bias_{\lambda }(n)=\frac{1}{N}\sum_{i=1}^{N}({\hat{\lambda }}_{i}-\lambda )\\ MSE_{\lambda }(n)=\frac{1}{N}\sum_{i=1}^{N}{\left({\hat{\lambda }}_{i}-\lambda \right)}^{2} \end{array}$$
  where *i* = 1, 2, 3, …, *N*.

**Fig 4:**
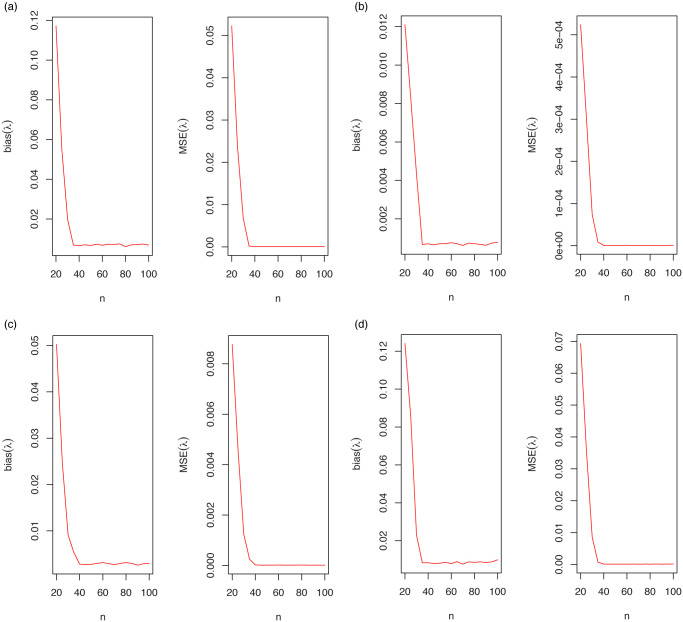
The bias and MSE for λ = 0.1.

The empirical results are graphically summarized in Fig 4. As seen from these results, the estimated biases and MSEs approach to zero for all parameter estimation methods. There are no clear differences between the performance of the estimation methods. However, the LSE method approach to desired values of the biases and MSEs faster than other estimation methods.

## 6 Empirical results

Two data sets are considered to show the flexibility of the OLiHL distribution against the several competitive models. We make the computational codes available at https://github.com/emrahaltun/Computational-codes-of-OLiHL-distribution.git.

### 6.1 Carbon fibers data

The fitting performance of the OLiHL distribution is compared with the below competitive models.

1. Exponentiated HL (EHL)
  $$\begin{array}{c} F_{\text{EHL}}\left(x;\lambda \right)={\left(\frac{1-e^{-x}}{1+e^{-x}}\right)}^{\lambda };\;x,\lambda >0, \end{array}$$
2. Generalized HL (GHL)
  $$\begin{array}{c} F_{\text{GHL}}\left(x;\lambda \right)=1-{\left(\frac{2e^{-x}}{1+e^{-x}}\right)}^{\lambda };\;x,\lambda >0, \end{array}$$
3. Lindley (Li)
  $$\begin{array}{c} F_{\text{Li}}\left(x;\lambda \right)=1-\left(1+\frac{\lambda x}{1+\lambda }\right)e^{-\lambda x};\;x,\lambda >0, \end{array}$$
4. Inverse Lindley (ILi)
  $$\begin{array}{c} F_{\text{ILi}}\left(x;\lambda \right)=\left(1+\frac{\lambda }{(1+\lambda )x}\right)e^{-\frac{\lambda }{x}};\;x,\lambda >0, \end{array}$$
5. Transmuted HL (THL)
  $$\begin{array}{c} \begin{array}{c} F_{THL}(x;\lambda )=\frac{\left(e^{x}-1)(1+2\lambda +e^{x}\right)}{{\left(1+e^{x}\right)}^{2}},x>0,\\ |\lambda |<1, \end{array} \end{array}$$ (23)
6. Exponential (Exp)
  $$\begin{array}{c} F_{Exp}(x;\lambda )=1-e^{-\lambda x},x>0,\lambda >0. \end{array}$$ (24)

We use the results of the Kolmogorov-Smirnov test (KS) with its p-value, Anderson-Darling (*A*^⋆^) and Cramér-von Mises (*W*^⋆^) as well as estimated −*ℓ* to decide the best-fitted model for the data used. The lowest value of the KS, *A*^⋆^ and *W*^⋆^ test statistics and the lowest value of −*ℓ* show the best-fitted model. The data set contains the breaking stress of carbon fibers. The number of observations is *n* = 66 and this data was reported by [32]. Table 1 contains the estimated parameter values of the all fitted model with asymptotic standard errors (SEs) as well as the goodness of fit statistics. The results in Table 1 reveal that the OLiHL could be chosen as the best model for the data used, since it has the lowest values of the goodness-of-fit statistics. The asymptotic confidence interval of the parameter λ of the OLiHL distribution is (0.139, 197).

**Table 1:** The estimated parameters of the fitted models with goodness-of-fit tests.

Distributions	Estimated parameters	SEs	−*ℓ*	A*	W*	KS	p-value
OLiHL	0.168	0.015	89.925	0.851	0.116	0.110	0.407
EHL	5.067	0.624	93.703	1.534	0.283	0.136	0.172
GHL	0.464	0.057	122.359	1.199	0.223	0.315	<0.001
THL	5.149×10^−8^	1.046×10^−5^	147.855	1.086	0.203	0.580	<0.001
Li	0.590	0.053	122.384	1.148	0.214	0.298	<0.001
ILi	2.891	0.296	134.669	4.070	0.721	0.370	<0.001
Exp	0.362	0.045	132.994	1.334	0.246	0.358	<0.001

Table 2 shows various estimation methods of the OLiHL parameter for the breaking stress of carbon fibers. The test statistics value of KS tests of the LSE, WLSE, and CVME methods are smaller than those of the MLE method for the OLiHL distribution. This result shows the fact that LSE, WLSE, and CVME methods could be more appropriate estimation methods than MLE for this data set. As mentioned in the simulation study, the LSE is a more appropriate method especially for small sample sizes which is consistent with the results obtained in this application.

**Table 2:** The results of the LSE, WLSE and CVME methods for the breaking stress of carbon fibers.

Statistic ↓ Method →	LSE	WLSE	CVME
λ	0.1909	0.1902	0.1911
KS	0.064	0.064	0.063
p-value	0.950	0.952	0.949

**Fig 5:**
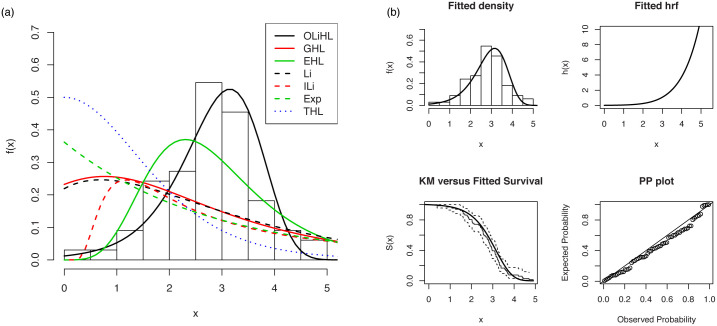
The fitted pdfs of the competitive models (top panel) and estimated functions of the OLiHL (bottom panel) distribution.

Fig 5(top) displays the fitted pdfs of the competitive models on the histogram of the data. As seen from these figures, the OLiHL distribution is the best model to describe the characteristics of the modelled data set. Fig 5(bottom) displays the fitted functions of the OLiHL distribution such as fitted pdf, cdf and survival functions with Kaplan-Meier (KM) curve as well as corresponding probability-probability (PP) plot. These figures also reveal that the OLiHL distribution provides superior fit to the modelled data set.

**Fig 6:**
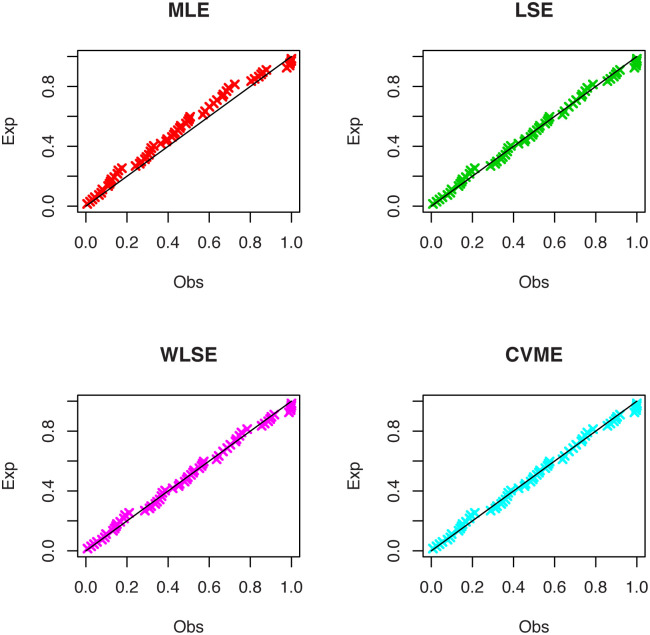
The PP plots of OLiHL distribution obtained under LSE, WLSE and CVME methods for the carbon fibers data set.

Fig 6 displays the PP plots of the OLiHL distribution obtained under the LSE, WLSE and CVME estimation methods. These estimation methods produce similar results since they are all based on the minimization of the distance between the empirical and theoretical distribution functions.

**Fig 7:**
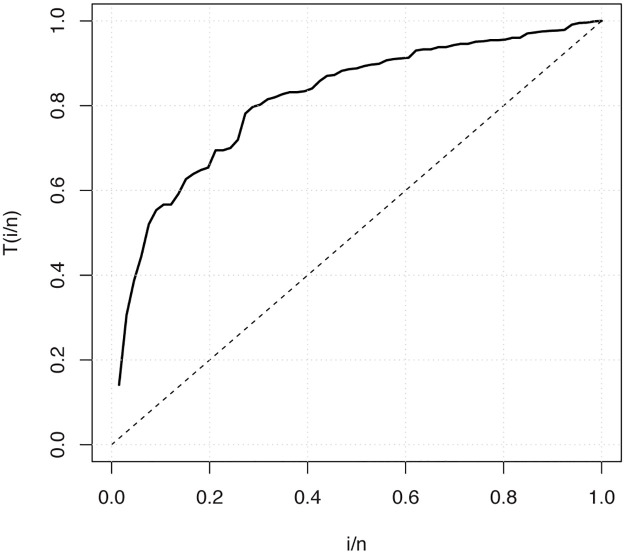
The TTT plots of the carbon fibers data set.

Finally, the total test time (TTT) plot, introduced by [33], is displayed in Fig 7 to see the empirical behavior of the hrf function. The TTT plot shows that the used data has increasing hrf which means that the OLiHL distribution can be used to model this data.

### 6.2 HIV+ data

We compare the performance of the LOLiHL regression model with log-exponential (LE) and log-Burr-Hatke-exponential (LBHE) regression models (see, [34], for details of LBHE and LE regression models). The modeled data set consists of 100 individuals having HIV+. The detailed information on the data set can be obtained from **Bolstad2** package of R software. The used data set was modeled by [34]. The depended variable, *y*_*i*_—observed survival time (in months) with censoring indicator *cens*_*i*_ (0 = alive, 1 = death), is modeled with two covariates: the history of drug use, *x*_*i*1_(1 = yes, 0 = no) and the ages of patients, *x*_*i*2_. The following model is fitted by LOLiHL, LBHE, and LE regression models.

$$\begin{array}{c} y_{i}=\beta_{0}+\beta_{1}x_{i1}+\beta_{2}x_{i2}+\sigma z_{i}. \end{array}$$

The above regression model is fitted by MLE and estimated parameter value, standard errors, the negative value of log-likelihood, and Akaike Information Criteria (AIC) are reported in Table 3. The AIC statistics is widely used to decide the best model among other competitive models (see [35, 36]). More information on the AIC statistics can be found in [37]. Since the LOLiHL regression model has the lowest value of AIC statistic, we conclude that the LOLiHL regression model produces a better fit than the other two regression model: LBHE and LE regression models. The regression parameters *β*_0_, *β*_1_ and *β*_2_ are found statistically significant 1% level. According to the estimated regression parameters, the individuals having drug use have lower lifetimes than non-drug use individuals. Moreover, when the ages of individuals increase, the lifetimes decrease.

**Table 3:** The estimated parameters of regression models and AIC statistic.

Parameters	L-E			LBHE			LOLiHL		
	Estimates	Sth. Errors	p values	Estimates	Sth. Errors	p values	Estimates	Sth. Errors	p values
λ	1.599	13.783	-	1.508	13.659	-	17.490	18.132	-
*σ*	0.839	0.072	-	0.778	0.067	-	0.991	0.114	-
*β*_0_	6.542	7.256	0.367	6.883	7.064	0.330	2.090	0.158	<0.001
*β*_1_	-0.091	0.014	<0.001	-0.091	0.014	<0.001	-0.015	0.004	<0.001
*β*_2_	-1.049	0.189	<0.001	-1.021	0.193	<0.001	-0.306	0.087	<0.001
−*ℓ*	128.502			128.059			125.527		
AIC	267.005			266.117			261.055		

**Fig 8:**
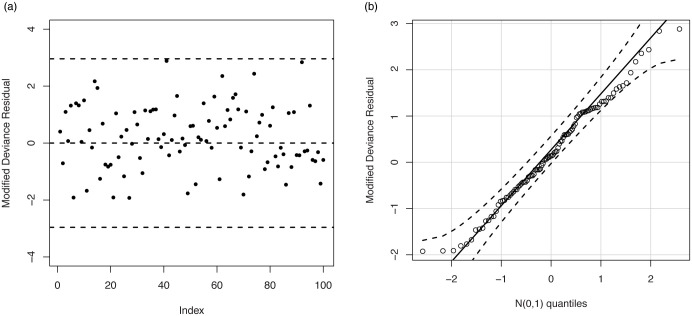
The plot of the modified deviance residual (a) and Q-Q plot of the modified deviance residual.

Fig 8 displays the results of the residual analysis for the LOLiHL regression model. As seen from Fig 8, there is no observation to be considered as possible outliers which reveals that the LOLiHL regression model provides an adequate fit to the used data set.

## 7 Discussions

The performance of the OLiHL distribution is compared with one-parameter competitive models. The OLiHL distribution has achieved to exhibit better modeling ability than Li, ILi, GHL and EHL distributions. Because the parameters of the Li and ILi distributions treat as location parameters. The location parameters do not affect the flexibility of the distribution. The parameter of the EHL distribution controls the shape of the distribution. However, the odd Lindley-G family provides more flexibility than the Exponentiated-G family. Therefore, OLiHL distribution works better than other competitive models.

## 8 Conclusion and future research

This study proposes a new one-parameter lifetime distribution, called as odd Lindley half-logistic distribution, shortly OLiHL distribution. The advantage of the OLiHL distribution is that it has only one parameter and this parameter controls the shape of the distribution which can be left-skewed, right-skewed, or nearly symmetric. This flexibility of the OLiHL distribution provides an opportunity to data scientists in modeling the different types of data sets. Additionally, the LOLiHL regression model will be a useful choice for practitioners studying in the field of survival analysis. As a future work of this study, we plan to develop the heteroscedastic regression model of the OLiHL distribution with its influence diagnostics and residuals analysis. We hope that the OLiHL distribution will find a wider application area in the near future.
